# ANTH domain-containing proteins are required for the pollen tube plasma membrane integrity via recycling ANXUR kinases

**DOI:** 10.1038/s42003-018-0158-8

**Published:** 2018-09-26

**Authors:** Keita Muro, Kumi Matsuura-Tokita, Ryoko Tsukamoto, Masahiro M. Kanaoka, Kazuo Ebine, Tetsuya Higashiyama, Akihiko Nakano, Takashi Ueda

**Affiliations:** 1 Department of Biological Sciences, Graduate School of Science, The University of Tokyo, Bunkyo-ku, Tokyo, 113-0033 Japan; 2 Division of Cellular Dynamics, National Institute for Basic Biology, Nishigonaka 38, Myodaiji, Okazaki, Aichi 444-8585 Japan; 3 Division of Biological Science, Graduate School of Science, Nagoya University, Furo-cho, Chikusa-ku, Nagoya, Aichi 464-8602 Japan; 4 Institute of Transformative Bio-Molecules (WPI-ITbM), Nagoya University, Furo-cho, Chikusa-ku, Nagoya, Aichi 464-8601 Japan; 5 Department of Basic Biology, SOKENDAI, Nishigonaka 38, Myodaiji, Okazaki, Aichi 444-8585 Japan; 6 Live Cell Super-Resolution Imaging Research Team, RIKEN Center for Advanced Photonics, 2-1 Hirosawa, Wako, Saitama 351-0198 Japan

## Abstract

During plant reproduction, sperm cells are delivered to ovules through growing pollen tubes. This process involves tip-localized receptor kinases regulating integrity and/or guidance of pollen tubes, whose localizations must be strictly regulated. However, the molecular basis for tip-localization of these molecules remains largely elusive. Here we show that a pair of AP180 N-terminal homology domain-containing proteins, PICALM5a and PICALM5b, is responsible for the tip-localization of ANXUR receptor kinases acting in an autocrine signaling pathway required for pollen tube integrity in *Arabidopsis thaliana*. The *picalm5a picalm5b* double mutant exhibits reduced fertility, and the double mutant pollen is defective in pollen tube integrity with premature bursts. The tip localization of ANXUR proteins is severely impaired in *picalm5a picalm5b* pollen tubes, whereas another receptor kinase PRK6 acting in pollen tube guidance is not affected. Based on these results, we propose that PICALM5 proteins serve as specific loading adaptors to recycle ANXUR proteins.

## Abstract (web summary)

Keita Muro et al. identify PICALM5a and PICALM5b as key proteins involved in pollen tube growth in *Arabidopsis thaliana*. Plants deficient in these two proteins exhibit decreased fertility, with impaired pollen tube integrity.

## Introduction

Plant cells internalize nutrients, proteins, and membrane materials via multiple endocytic pathways, wherein clathrin-mediated endocytosis (CME) is responsible for major endocytic activities^1^. Pollen tube growth, a key event in sexual plant reproduction, also depends on CME^2,3^, whose molecular basis, however, remains largely elusive. In mammals, AP180 N-terminal homology (ANTH) domain-containing proteins are proposed to mediate clathrin-coated pit formation and cargo sorting at the plasma membrane during CME by binding with phosphoinositides, clathrin, and cargo proteins^4^. ANTH domain-containing proteins (ANTH proteins) are also conserved in plants. The *Arabidopsis thaliana* genome encodes 18 ANTH proteins (Supplementary Table 1), whereas metazoan and fungal genomes containing fewer, implying that plant cell ANTH proteins have more divergent functions than non-plant systems^5^. Some ANTH proteins in *Arabidopsis* are localized to the plasma membrane, endosomes, and cell plates^6^. Phosphatidylinositol binding clathrin assembly protein 4a (PICALM4a) and PICALM4b (aka CAP1) have been shown to directly interact with TML, a core component of the TPLATE/TSET complex^7^, and PICALM6/AP180 is shown to localize at the subapical plasma membrane in tobacco pollen tubes^8^. These results suggest that ANTH proteins have roles in plant endocytic processes. However, the precise molecular and physiological functions of ANTH proteins have not been elucidated in plants.

During fertilization, pollen tubes reach ovules by growing very rapidly in one direction^9^. In growing pollen tubes, secretory vesicles are delivered to an inverted cone-shaped region in the tip to supply cell wall and plasma membrane materials^10–13^. Meanwhile, excessive plasma membrane materials are sequestered via endocytosis^13–16^. Studies on endocytosis in growing pollen tubes using the endocytic tracer FM4-64 suggested that most endocytosed materials were rapidly recycled to the secretory pathway^17^. Pollen tube CME occurs mainly in the subapical region of the plasma membrane^13,18^, which is supported by the fact that clathrin coat components accumulate in this region^8,19^. However, the molecular mechanisms that sort specific cargo into clathrin-coated vesicles and their physiological significance have not yet been elucidated. Recent studies have also identified tip-localized receptor-like kinases required for pollen tube growth, which include PRK6, ANXUR1/2, and BUPS1/2, the receptors responsible for pollen tube guidance^20^ or pollen tube integrity^21–24^. The tip localization and plasma membrane amounts of these receptors must be strictly regulated, which should be accomplished by tightly regulated exocytic and endocytic trafficking activities. However, machinery components mediating the tip localization of these receptors have not yet been identified.

Here, we report the essential roles of the redundantly functioning ANTH domain-containing proteins in tip-localization of ANXUR1/2 and sustained pollen tube integrity by probably acting as specific loading adaptors during CME.

## Results

### PICALM5a and PICALM5b are required for male fertility

**Fig. 1:**
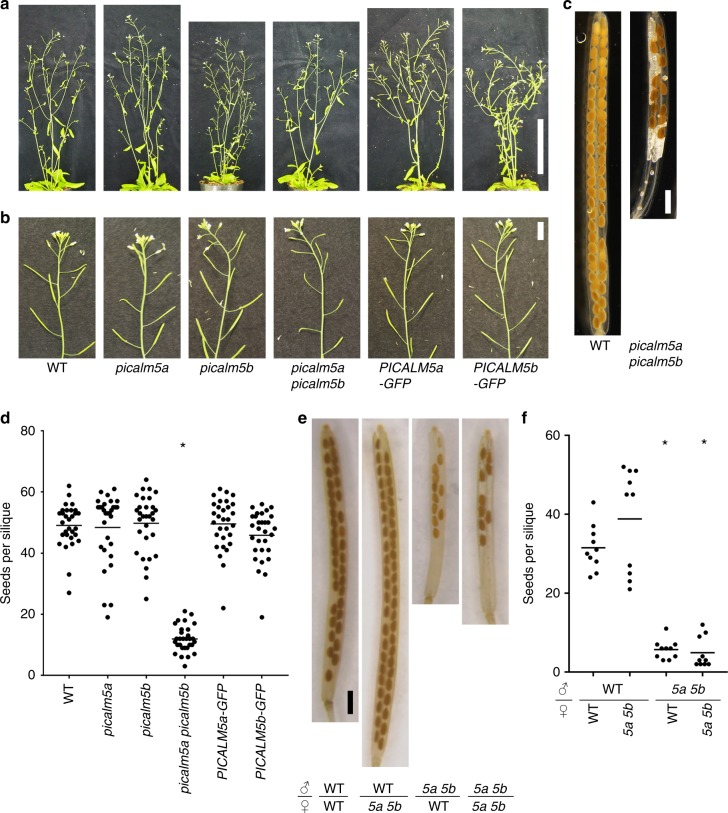
The *picalm5a picalm5b* double mutation leads to reduced male fertility. **a** and **b** Shoots (**a**) and inflorescences (**b**) of six-week-old wild-type (WT), *picalm5a*, *picalm5b*, *picalm5a picalm5b*, *PICALM5a-GFP* in *picalm5a picalm5b*, and *PICALM5b-GFP* in *picalm5a picalm5b* plants. Scale bars = 10 cm (**a**) and 1 cm (**b**). **c** Representative cleared siliques of self-pollinated WT and *picalm5a picalm5b* plants. Scale bar = 1 mm. **d** Quantification of seeds per silique for wild-type, *picalm5a, picalm5b, picalm5a picalm5b, PICALM5a-GFP* in *picalm5a picalm5b*, and *PICALM5b-GFP* in *picalm5a picalm5b* plants (*n* = 30). Bars represent the means and the asterisk indicates a significant difference from the wild-type plant according to Welch’s *t* test (*p* = 3.04 × 10^−28^). **e** Representative cleared siliques obtained by cross pollination between parents with the indicated genotypes. *5a 5b* represents the *picalm5a picalm5b* double mutant. Scale bar = 1 mm. **f** Quantification of the seeds per silique for cross-pollinated siliques (*n* = 10 siliques for each combination). *5a 5b* represents the *picalm5a picalm5b* double mutant. Bars represent the means and asterisks indicate a significant difference from the value for crosses between male and female wild-type plants according to Welch’s *t* test (*p* = 1.80 × 10^-8^ for cross between male 5a 5b and female wild-type plants and 1.72 × 10^-9^ for male and female 5a 5b plants)

Functional redundancy in *Arabidopsis* ANTH proteins might explain why the loss-of-function effects of these genes have not been reported to date. Therefore, to elucidate the physiological significance of ANTH proteins in pollen tube growth, we began our study by generating multiple ANTH protein mutants by crossing T-DNA insertion mutants of ANTH proteins to obtain abnormal fertility phenotypes. Among the multiple mutants we generated, the double mutant of the closely related genes^5^ *PICALM5a* (aka *ECA2*, At1g03050) and *PICALM5b* (At4g02650), exhibited marked fertility abnormality (Fig. 1 and Supplementary Fig. 1). Although the vegetative growth of *picalm5a picalm5b* plants was indistinguishable from that of wild-type and the single mutant plants, their siliques were significantly shorter than those produced by wild-type plants (Fig. 1a, b). We then cleared these siliques to observe the seeds inside, and found that the number of seeds contained in the mutant siliques was significantly reduced compared to that in wild-type plants (Fig. 1c, d, *p* = 3.04 × 10^−28^ by Welch’s *t* test).

We performed reciprocal cross pollination between the wild-type and *picalm5a picalm5b* double mutant plants to investigate whether this phenotype resulted from a defect in the male or female function (Fig. 1e, f). When pistils of the *picalm5a picalm5b* mutant were pollinated with wild-type pollen grains, silique lengths and number of seeds were comparable to those of the self-pollinated wild-type plant. Conversely, when pistils of the wild-type plants were pollinated with pollen grains from the *picalm5a picalm5b* mutant, siliques were shorter and fewer seeds were generated. These results indicate that the *picalm5a picalm5b* mutation leads to defective pollen function, which reduces fertility.

### PICALM5 is required for pollen tube integrity

Mature double mutant pollen grains were not morphologically distinguishable from wild-type pollen when pollen grains produced on the *picalm5a picalm5b* and wild-type plants were observed with a scanning electron microscope (Supplementary Fig. 2a) or stained with DAPI (Supplementary Fig. 2b). Moreover, we did not detect difference in the viability of these pollen grains visualized with fluorescein diacetate (FDA) and propidium iodide (Supplementary Fig. 2c). These suggested that pollen successfully developed in the *picalm5a picalm5b* mutant.

**Fig. 2:**
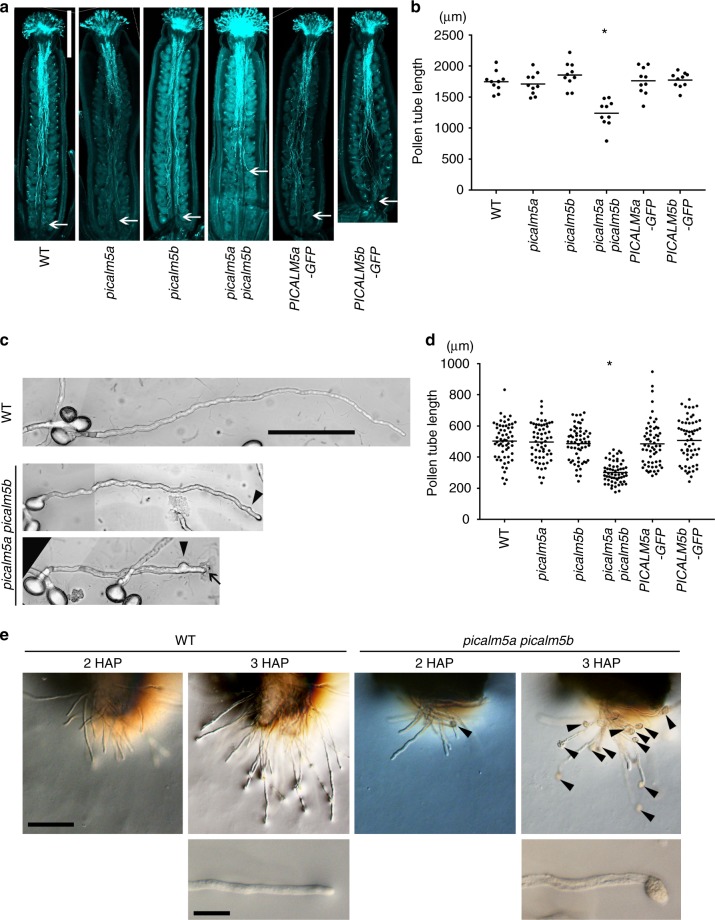
PICALM5a and PICALM5b are essential for sustained pollen tube growth. **a** Aniline blue staining of pistils from wild-type, *picalm5a*, *picalm5b, picalm5a picalm5b, PICALM5a-GFP* in *picalm5a picalm5b*, and *PICALM5b-GFP* in *picalm5a picalm5b* plants. Pistils were harvested 12 h after hand pollination. The white arrows indicate the tips of the longest pollen tubes. Scale bar = 500 μm. **b** Quantification of lengths of the longest pollen tubes in pistils 12 h after pollination (*n* = 10 pistils). Bars represent means and the asterisk indicates a significant difference from the result of the wild type according to Welch’s *t* test (*p* = 1.64 × 10^-5^). **c** Wild-type (WT) and *picalm5a picalm5b* pollen tubes grown in vitro. Arrow heads and an arrow indicate knotted structure and burst tip of *picalm5a picalm5b* pollen tubes. Scale bar = 100 μm. **d** Quantification of wild-type, *picalm5a*, *picalm5b, picalm5a picalm5b, PICALM5a-GFP* in *picalm5a picalm5b*, and *PICALM5b-GFP* in *picalm5a picalm5b* pollen tube lengths after 5 h of incubation (*n* = 60). Bars represent means and the asterisk indicates a significant difference from the WT pollen tubes according to Welch’s *t* test (*p* = 3.84 × 10^-19^). **e** WT and *picalm5a picalm5b* pollen tubes grown semi-in vivo 2 and 3 h after pollination. Magnified pollen tube tips are also shown for pollen tube tips 3 h after pollination. Arrowheads indicate burst pollen tubes. Scale bars = 100 μm and 20 μm for low and high magnification images, respectively. HAP hours after pollination

In the observation of *picalm5* mutant siliques, we noticed that seeds were formed on only the apical side of the *picalm5a picalm5b* double mutant siliques (Fig. 1c, e). The seed distribution suggested that fertilization succeeded on only the apical part of the pistil, which was possibly due to limited pollen tube growth in pistils. To verify this possibility, we examined pollen tube elongation in vivo. Hand-pollinated wild-type and *picalm5a picalm5b* pistils were stained using aniline blue 12 h after pollination. The *picalm5a picalm5b* double mutant pollen tubes were significantly shorter than wild-type and single mutant pollen tubes (*p* = 1.64 × 10^−5^ by Welch’s *t* test). Furthermore, the double mutant pollen tubes were unable to reach the ovules in the basal part of the pistils (Fig. 2a, b).

We also examined pollen tube elongation in vitro. The *picalm5a picalm5b* double mutant pollen tubes were shorter than the wild-type pollen tubes, and knotted pollen tubes were frequently observed, whereas the lengths and morphologies of *picalm5a* and *picalm5b* single mutant pollen tubes were similar to those of wild-type pollen tubes (Fig. 2c, d). Furthermore, the majority (64%) of pollen tube tips in the *picalm5a picalm5b* double mutant burst and released their cytoplasm during incubation, whereas less than 5% of wild-type or *picalm5* single mutant pollen tubes burst during 5-h in vitro incubation (*n* = 333–429 for each genotype) (Fig. 2c, Supplementary Fig. 3). We also performed a semi-in vivo pollen tube growth assay. Both wild-type and *picalm5a picalm5b* double mutant pollen tubes began to emerge from the cut end of the style until 2 h after pollination. While none of wild-type pollen tubes burst 3 h after pollination (*n* = 35), 85.6 % of double mutant pollen tubes burst at this time point (*n* = 99) (Fig. 2e). These results indicate that PICALM5a and PICALM5b are required for pollen tube integrity, which is essential for fertilization in the basal part of the pistil.

### Localization of PICALM5 in pollen tubes

**Fig. 3:**
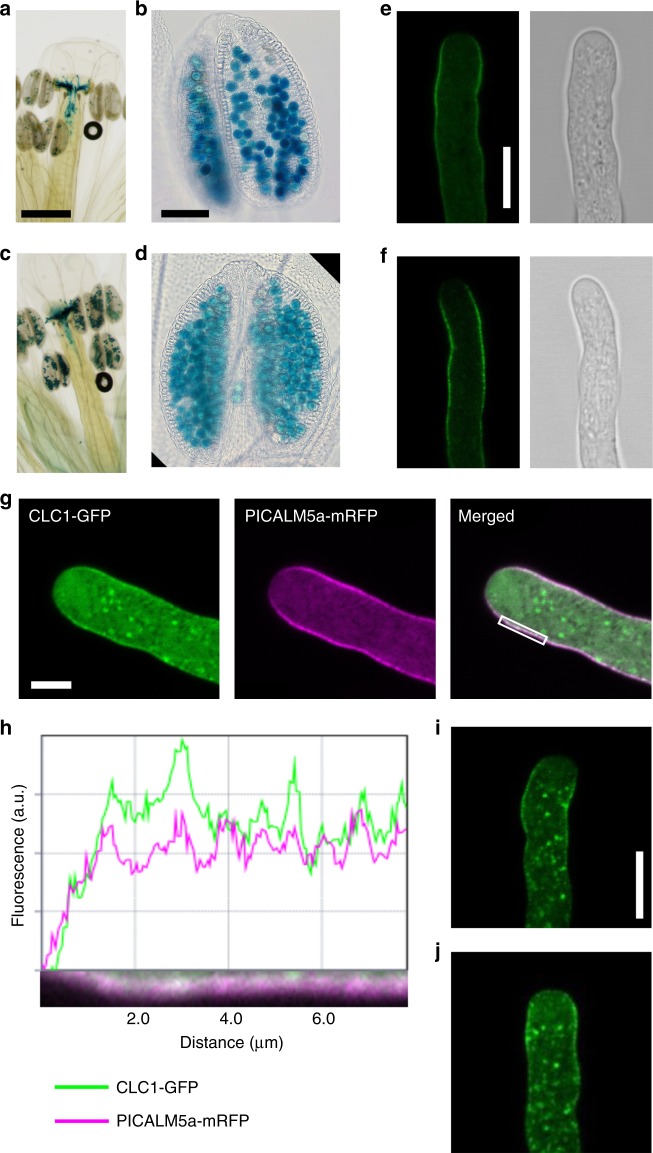
Localization of PICALM5a and PICALM5b in growing pollen tubes. **a**–**d** GUS staining of pistils (**a**, **c**) and anthers (**b**, **d**) from *PICALM5apro:GUS* (**a**, **b**) and *PICALM5bpro:GUS* (**c**, **d**) plants. Scale bars = 500 μm (**a**, **c**) and 100 μm (**b**, **d**). **e**, **f** Subcellular localization of PICALM5a-GFP (**e**) and PICALM5b-GFP (**f**) in pollen tubes from the *picalm5a* and *picalm5b* mutants germinated in vitro. Scale bar = 10 μm. The data are representatives of more than ten pollen tubes observed for each transgenic line. **g** Subcellular localizations of CLC1-GFP and PICALM5a-mRFP in a wild-type pollen tube germinated in vitro. Scale bar = 5 μm. The data are representatives of more than 12 pollen tubes observed. **h** Fluorescence intensity profiles for the white box shown in **g**. **i**, **j** Subcellular localizations of CLC1-GFP in a growing pollen tube from a wild-type (**i**) plant and a *picalm5a picalm5b* (**j**) plant germinated in vitro. Scale bar = 10 μm. The representative data of more than ten pollen tubes observed for each transgenic line are presented

According to the public microarray database^25,26^, *PICALM5a* and *PICALM5b* are mainly expressed in mature pollen grains and germinated pollen tubes (Supplementary Fig. 4). To confirm this expression pattern, we performed a promoter reporter assay by generating transgenic plants expressing β-glucuronidase under the regulation of *PICALM5a* or *PICALM5b* promoters in wild-type Arabidopsis plants (*PICALM5a*_*pro*_*:GUS* and *PICALM5b*_*pro*_*:GUS*). GUS signals were mainly observed in mature pollen grains and pollen tubes growing in the pistils of both transgenic plant types (Fig. 3a–d), which was consistent with the microarray data.

We then expressed GFP-tagged PICALM5a and PICALM5b in the *picalm5a picalm5b* double mutant. These constructs rescued the defective pollen tube growth associated with premature rupture of the double mutant in vitro and in vivo and increased the fertilization efficiency and silique growth (Figs. 1 a, b, d and 2a, b, d, Supplementary Fig. 3). The results confirmed that these phenotypes were caused by *PICALM5a* and *PICALM5b* loss-of-function, and demonstrated the functionality of the GFP-tagged PICALM5 proteins.

We then observed the subcellular localizations of PICALM5a-GFP and PICALM5b-GFP expressed in their respective single mutants. According to the definition of regions in growing pollen tubes by Chebli et al.^27^, both fluorescent-tagged PICALM5 proteins were mainly localized to the subapical plasma membrane region of the growing pollen tubes (Fig. 3e, f). To verify a role of PICALM5 in CME, we then examined whether the PICALM5a protein co-localized with clathrin in a transgenic plant co-expressing GFP-fused clathrin light chain 1 (CLC1-GFP) and mRFP-tagged PICALM5a. We chose CLC1 for this analysis because *CLC1* is expressed most abundantly in pollen among three clathrin light chain members in *Arabidopsis*^25,26^, and a mutation in *CLC1* results in severe defect in pollen viability^28^. The immunofluorescence analysis also showed that CLC presents at punctate structures at the cytoplasm and the plasma membrane in the subapical region of pollen tubes^19^. CLC1-GFP was mainly observed at punctate cytoplasmic structures and the subapical plasma membrane in the growing pollen tubes and colocalized with PICALM5a-mRFP on the plasma membrane (Fig. 3g). The co-localization was especially evident at punctate foci in the subapical plasma membrane, which was further demonstrated by the quantified fluorescence intensity (Fig. 3h and Supplementary Fig. 5). These results suggest that PICALM5 proteins are involved in CME on the subapical plasma membrane of growing pollen tubes.

### PICALM5 mediates tip-localization of ANXUR receptor kinases

**Fig. 4:**
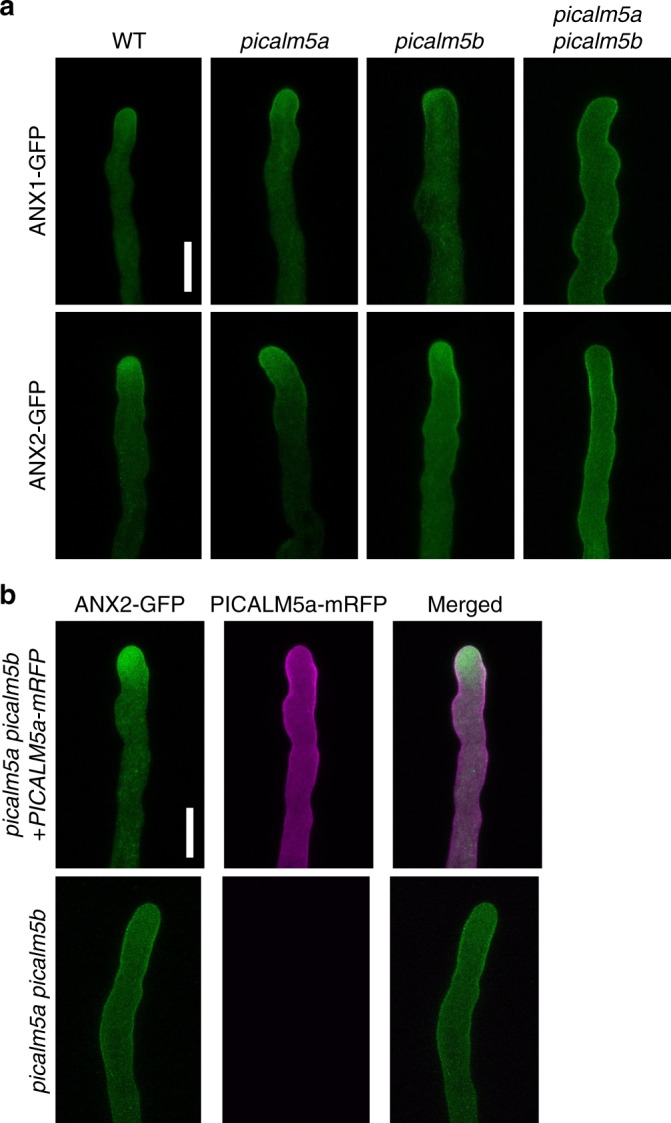
PICALM5a and PICALM5b are required for the correct localization of ANXUR receptor-like kinases. **a** Subcellular localizations of ANX1-GFP and ANX2-GFP in growing pollen tubes of wild-type, *picalm5a*, *picalm5b*, and *picalm5a picalm5b* plants germinated in vitro. Scale bar = 10 μm. The data are representatives of more than seven pollen tubes observed for each transgenic line. **b** Subcellular localizations of ANX2-GFP in in vitro-germinated growing pollen tubes of transgenic plants harboring a single copy of *ANX2-GFP* expressed with or without *PICALM5a-mRFP* on the *picalm5a picalm5b* background. Scale bar = 10 μm. The data are representatives of six (with *PICALM5a-mRFP*) or four (without *PICALM5a-mRFP*) pollen tubes observed, respectively

We then observed CLC1-GFP in pollen tubes of the *picalm5a picalm5b* mutant but found no marked effect on its distribution (Fig. 3i, j). Therefore, if any effects of PICALM5a and PICALM5b absences on general CME exist, they are subtle. Thus, we speculated that the *picalm5a picalm5b* pollen tube elongation defect may result from the disordered transport of specific proteins necessary for proper pollen tube elongation. To identify such proteins, whose transport is mediated by PICALM5 proteins, we searched for plasma membrane proteins whose impairments result in pollen tube defects similar to those in the *picalm5a picalm5b* mutant. Pollen tubes with defective ANXUR (ANX) 1 and ANX2 receptor kinases, which redundantly regulate pollen tube integrity via an autocrine signaling pathway^23,24^, are shortened, knotted, and associated with premature rupture^21,22^. The similar pollen tube phenotypes for the *anx* and *picalm5* mutations prompted us to evaluate the localization of the ANX proteins in the *picalm5a picalm5b* double mutant. Intriguingly, ANX1-GFP and ANX2-GFP exhibited remarkably different localizations between the wild-type and *picalm5a picalm5b* plants (Fig. 4a). In the wild-type pollen tubes, tip-focused localizations were observed for ANX1-GFP and ANX2-GFP. However, in the *picalm5a picalm5b* mutant, these proteins were localized to the subapical and distal plasma membrane and the cytoplasm without a tip-enriched signal. This localization pattern suggested that the *picalm5a picalm5b* mutant failed to trigger relocalization of ANX1 and ANX2 to the apical region of the pollen tubes, which led to broadened ANX localization on the plasma membrane. When expressed in the *picalm5a* and *picalm5b* single mutants, ANX1-GFP and ANX2-GFP showed subcellular localizations similar to those in wild-type plants, which indicated the redundant functions of PICALM5a and PICALM5b in the localization of ANX proteins. Mislocalization of ANX2-GFP in pollen tubes and the reduced seed number in *picalm5a picalm5b* were rescued by the expression of *PICALM5a-mRFP*, which further indicated that PICALM5 proteins are responsible for the proper localization of ANX receptor kinases as well as the fertility (Fig. 4b, Supplementary Fig. 6). Intriguingly, expression of *ANX1-GFP* or *ANX2-GFP* partially but significantly suppressed the fertility defect of the *picalm5a picalm5b* plant (Supplementary Fig. 6b, *p* = 7.83 × 10^−4^ and 3.29 × 10^−10^ by Welch’s *t* test for *ANX1-GFP* and *ANX2-GFP*, respectively). This genetic interaction supports the notion that the deleterious effect of the *picalm5a picalm5b* double mutations on fertility is attributed to faulty functions of ANX proteins in the double mutant.

**Fig. 5:**
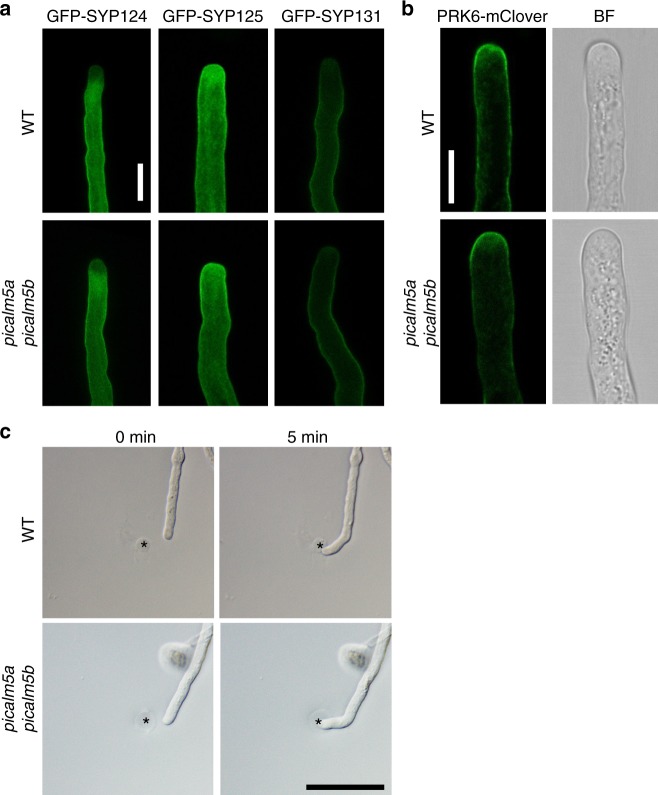
PICALM5a and PICALM5b are not required for the correct localization of SYP1 Qa-SNAREs and PRK6 receptor-like kinase. **a** Subcellular localizations of GFP-SYP124, GFP-SYP125, and GFP-SYP131 in growing pollen tubes of wild-type (WT) and *picalm5a picalm5b* plants germinated in vitro. Scale bar = 10 μm. The data are representatives of more than ten pollen tubes observed for each transgenic line. **b** Subcellular localizations of PRK6-mClover in growing pollen tubes of WT and *picalm5a picalm5b* plants germinated in vitro. Bright-field (BF) images are also shown. Scale bar = 10 μm. The data are representatives of five pollen tubes observed for each transgenic line. **c** WT and *picalm5a picalm5b* pollen tubes just after (0 min) and 5 min after application of AtLURE1.2-containing gelatin beads. Asterisks indicate AtLURE1.2-containing gelatin beads. Scale bar = 50 μm. Both wild-type and *picalm5a picalm5b* pollen tubes were attracted to AtLURE1.2

The effect of *picalm5* mutations is specific to ANX proteins. When we assessed the localization of GFP-tagged SYP124, SYP125, and SYP131, other plasma membrane proteins with distinctive localization patterns on the pollen tube plasma membrane^29,30^, no notable differences in their localizations between mutant and wild-type pollen tubes were observed (Fig. 5a). Furthermore, tip-localization of another receptor kinase, PRK6, which is responsible for pollen tube guidance by the LURE ligand^20^, was not markedly affected by the *picalm5a picalm5b* mutation (Fig. 5b). A pollen tube attraction assay further demonstrated the intact PRK6-mediated signaling in the *picalm5a picalm5b* mutant; 93.3% of the *picalm5a picalm5b* double mutant pollen tubes (*n* = 15) were attracted to AtLURE1.2 prior to premature burst, which was comparable to the rate of attracted pollen tubes of wild type (100%, *n* = 15) (Fig. 5c). Thus, PICALM5a and PICALM5b are specifically required for the correct localization of ANX proteins.

## Discussion

Our results suggest that PICALM5a and PICALM5b are required for tip-localization of ANX proteins, which could reflect the function of the PICALM5 proteins as loading adaptors for ANX proteins in CME. Tip-localized receptor kinase PRK6 and plasma membrane-localized SNARE proteins were not affected by the *picalm5* double mutation. This result indicates that these receptor-like kinases are under the regulation of distinct trafficking/recycling mechanisms, which also indicates that PICALM5 proteins mediate CME of a specific set of plasma membrane proteins including ANX proteins. In a consistent manner, pollen tubes of the *picalm5* double mutant were normally attracted by AtLURE1.2, suggesting that the LURE-PRK6 signaling is not impaired by the *picalm5* double mutation.

Pollen tubes produced by the *anx1 anx2* double mutant rupture shortly after germination^21^. Conversely, pollen tubes produced by *picalm5a picalm5b* burst after growing to some extent. This phenotypic difference is most likely because de novo-synthesized ANX proteins transported to the tip region of the *picalm5* mutant can fulfill their functions during the early period of pollen tube growth, whereas during later stages there are not enough ANX proteins recycled back from the subapical region to sustain pollen tube integrity because of the absence of these specific adaptors.

ANX protein levels at the tip must be tightly regulated because over-accumulation of ANX proteins also results in defective pollen tube growth^31^, and our finding demonstrates that a specific set of ANTH proteins is required for the ANX-mediated maintenance of pollen tube integrity. Fertilization mediated by pollen tubes is a trait acquired relatively late during land plant evolution, and close homologs of PICALM5a and PICALM5b do not exist in basal land plants, such as lycophytes and bryophytes^5^, which do not show pollen tube-mediated delivery of male gametes. Therefore, rapid diversification of the ANTH protein group during land plant evolution could be associated with the acquisition of new plant functions, including pollen tube growth. During revision of this manuscript, another ANTH protein, PICALM9b/EAP1, was also reported to be involved in pollen tube growth^32^, although its involvement in endocytic recycling of plasma membrane proteins remains to be verified. Given the diverged structures of PICALM5 and PICALM9b, these proteins could be involved in CME of different plasma membrane proteins. Further identification of cargo proteins recognized by these adaptor proteins would be effective to unravel a molecular basis of functional diversification of ANTH domain-containing proteins involved in pollen tube growth, and additional studies of other ANTH proteins are also needed to elucidate the relationship between the diversification of endocytic mechanisms and the evolution of plant physiology.

## Methods

### Plant materials and growth conditions

All *A. thaliana* plants used in this study were on a Col-0 accession background. Seeds were grown on Murashige and Skoog (MS) agar medium containing 0.2% sucrose at 23 °C under continuous light. Two-week-old plants were transplanted into soil and grown at 23 °C under long-day light-dark cycles (16 h light and 8 h dark).

### Nomenclature

ANTH proteins in *Arabidopsis* were renamed. They are listed in Supplementary Table 1, and the names follow those established by De Craene et al.^33^ and Zouhar and Sauer^5^.

### Plasmid construction

To generate *PICALM5a-GFP*, *PICALM5a-mRFP, PICALM5b-GFP*, *GFP-SYP124*, *GFP-SYP125*, and *GFP-SYP131*, approximately 2 kb of upstream sequences and 1 kb of downstream sequences for the coding regions of each gene were PCR-amplified with the primers listed in Supplementary Table 2. The amplified fragments were then cloned into the pENTR/D-TOPO entry vector (Thermo Scientific). The clones were amplified by inverted PCR and combined with cDNA for GFP or mRFP using an In-Fusion HD Cloning Kit (Clontech).

To generate *ANX1-GFP* and *ANX2-GFP*, PCR-amplified genomic fragments containing approximately 2 kb of promoter sequences and coding regions for *ANX1* and *ANX2* were cloned into the pENTR/D-TOPO entry vector. The clones were recombined with the pGWB4 vector^34^ using an LR Clonase II enzyme mix (Thermo Scientific).

To generate *LAT52p:CLC1-GFP*, a genomic fragment containing the coding region for *CLC1* without the stop codon was PCR-amplified and subcloned into the SpeI site of the *YMv036* vector^35^ using an In-Fusion HD Cloning Kit (Clontech). PRK6-mClover was described previously^20^.

### Pollination and silique clearing

Buds that were about to open were emasculated one day before pollination. Cross-pollinated siliques were harvested 10–14 days after hand pollination and fixed/decolorized overnight in an ethanol:acetic acid solution (6:1). Before observation, the siliques were cleared with a chloral hydrate solution (8 g of chloral hydrate, 1 mL of glycerol, 2 mL of water). Images were obtained using a Leica MZ16 FA fluorescence stereomicroscope.

### DAPI staining

DAPI staining was performed as described by Park et al.^36^. Five to six open flowers were soaked in 300 μL of DAPI staining solution (0.4 μg/mL DAPI, 0.1% Triton X-100, 1 mM EDTA, 0.1 M NaPO_4_, pH 7.0) in microtubes. After briefly vortexing the samples, flower debris was removed with a pair of tweezers, and the microtubes were centrifuged to spin down the pollen grains. Pollen grains were transferred to a microscope slide and observed under a Zeiss LSM780 confocal microscope excited with UV.

### Scanning electron microscopy

Images of fresh pollen grains were obtained using the Hitachi TM-1000 tabletop scanning electron microscope.

### PI/FDA staining

Anthers from open flowers were soaked in PI/FDA staining solution (1 μM propidium iodide and 2.5 μM FDA) on glass slides. Released pollen grains were observed under an Olympus BX60 microscope with the NIBA filter for FDA and the WIG filter for PI.

### Aniline blue staining

Aniline blue staining was performed as described by Kaya et al.^37^. The pistils were harvested 12 h after hand pollination and fixed overnight in an acetic acid:ethanol solution (1:3) at room temperature. The fixed pistils were softened in 1 N NaOH for 30 min at 60 °C. After three rinses with 2% K_3_PO_4_, the pistils were stained with 0.01% aniline blue in 2% K_3_PO_4_ for 2 to 4 h in the dark. Fluorescence images were obtained using a Zeiss LSM780 inverted confocal microscope.

### GUS staining

The inflorescences were fixed in 90% acetone on ice for 15 min. The fixed inflorescences were briefly washed twice with 100 mM NaPO_4_ (pH 7.0) and placed into GUS staining solution (0.5 mg/mL X-Gluc, 1 mM potassium ferricyanide/ferrocyanide, 0.1% Triton X-100, 10 mM EDTA, and 100 mM NaPO_4_, pH 7.0). The samples in the GUS staining solution were vacuum-infiltrated for 15 min and incubated for 3 to 5 h at 37 °C. The stained samples were washed with 70% ethanol and decolorized with an ethanol:acetic acid solution (6:1).

### In vitro pollen germination

In vitro pollen germination was carried out essentially as described by Boavida and McCormick^38^ with some modifications. Pollen grains were applied to a cellulose cellophane sheet (Futamura Chemical)^39^ placed on a thin layer of pollen tube germination medium (0.01% H_3_BO_3_, 5 mM CaCl_2_, 5 mM KCl, 1 mM MgSO_4_, 10% sucrose, pH 7.5, 1.5% low-melting agarose) supplemented with 10 μM epibrassinolide (Sigma-Aldrich)^40^ on coverslips and incubated at 23 °C in a humid chamber. Images were obtained using an Olympus CKX53 inverted microscope, and pollen tube lengths were measured using ImageJ software (National Institutes of Health).

### Semi-in vivo pollen tube growth assay

Flowers were emasculated one day before the assay. Hand-pollinated pistils were cut at the junction of style and ovary to remove the ovary. The cut pistils were placed on pollen tube germination medium (0.001% H_3_BO_3_, 1.27 mM Ca(NO_3_)_2_, 0.4 mM MgSO_4_, 14% sucrose, pH 7.0, 1.5% low-melting agarose)^20^ and incubated at 22 °C. Numbers of burst pollen tubes were counted at 3 h after pollination. Pictures were taken with the Olympus IX73 inverted microscope equipped with DP73 digital camera (Olympus, Japan).

### Semi-in vivo pollen tube attraction assay

The semi-in vivo pollen tube attraction assay was performed as described previously^20^. Gelatin beads containing 10 μM of His-tagged AtLURE1.2 beads were placed in front of a pollen tube, which grew through the cut style. Because most of the double mutant pollen tubes burst 1 h after emergence from the cut-end of the style (see results of semi-in vivo pollen tube growth assay), the guidance assay was performed before they burst, approximately between 2 and 3 h after pollination. Pictures were taken with the Olympus IX73 inverted microscope equipped with DP73 digital camera (Olympus, Japan).

### Fluorescent protein imaging

Transgenic pollen grains were germinated in liquid pollen tube germination medium (0.01% H_3_BO_3_, 5 mM CaCl_2_, 5 mM KCl, 1 mM MgSO_4_, 10% sucrose, pH 7.5) supplemented with 10 μM epibrassinolide. After incubation for 5 to 7 h at 23 °C, fluorescent images were captured using a Zeiss LSM780 inverted confocal microscope.

### Statistics

To compare the lengths of pollen tubes germinated in vitro, we performed three independent experiments. The lengths of 20 pollen tubes were measured for each experiment and genotype. Statistical comparisons were performed using Welch’s *t* test, and a statistically significant difference (*p* = 3.84 × 10^−19^) is indicated with an asterisk.

In the fertility analysis, the number of seeds per silique was counted in 29–30 self-pollinated siliques or ten hand-pollinated siliques for each genotype. Statistical comparisons were performed using Welch’s *t* test, and statistically significant differences (*p* < 0.01) are indicated with an asterisk.

To compare the bursting rate of pollen tubes germinated in vitro, we performed three independent experiments. The tips of at least 97 pollen tubes were observed for each genotype in each experiment. Statistical comparisons were performed using Welch’s *t* test, and a statistically significant difference (*p* = 0.0196) is indicated with an asterisk.

The colocalization between CLC1-GFP and PICALM5a-mRFP was quantified in five pollen tubes using ImageJ using the pearson-spearman correlation colocalization plugin (The University of Nottingham, https://www.cpib.ac.uk/tools-resources/software/psc-colocalization-plugin/). Statistical comparison was performed using Student’s *t* test, and a statistically significant difference (*p* = 1.03 × 10^-6^) is indicated with an asterisk.

## Electronic supplementary material

Supplementary Information

## Data Availability

All data supporting the findings of this study are available within the article and its supplementary information. The materials from this study are available from the corresponding author on reasonable request.
