# Supplementary material for: ANTH domain-containing proteins are required for the pollen tube plasma membrane integrity via recycling ANXUR kinases
Source: Commun Biol. 2018 Sep 26;1:152. doi: 10.1038/s42003-018-0158-8 (PMC6158268; doi:10.1038/s42003-018-0158-8)
Supplement: Supplementary file 1 — Supplementary Information [file 42003_2018_158_MOESM1_ESM.pdf]

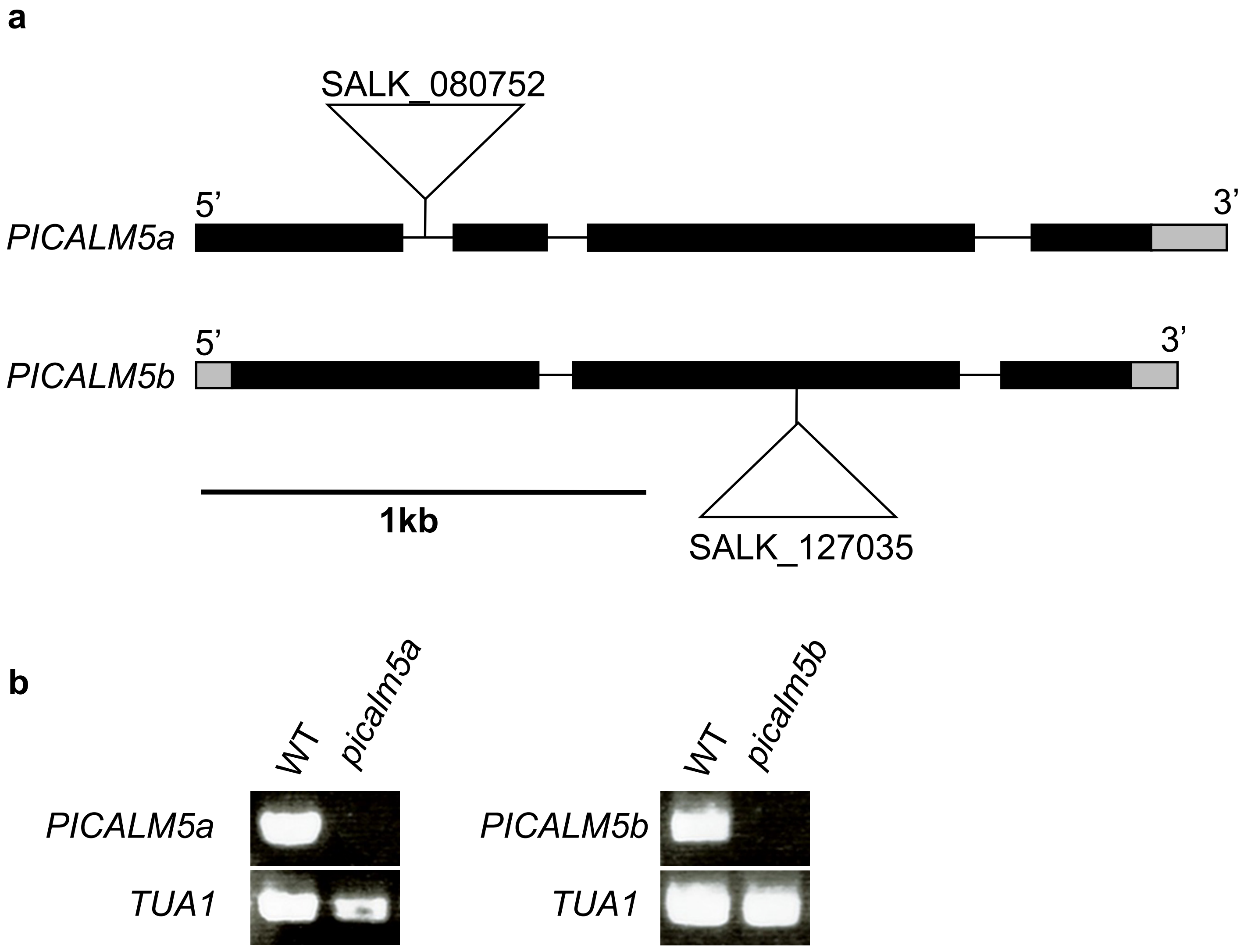

Supplementary Figure 1. The *picalm5* mutants used in this study. **a** Schematic structures of *PICALM5a* and *PICALM5b* genes with locations of the T-DNA insertions. Exons and 5’- and 3’-flanking regions are indicated with black and gray boxes, respectively. **b** A RT-PCR analysis of full-length transcripts of *PICALM5a* and *PICALM5b* in flowers of wild type (WT) and mutant plants. *TUA1* was used as a control. Full scan of the gel images are in Supplementary Fig. 7.

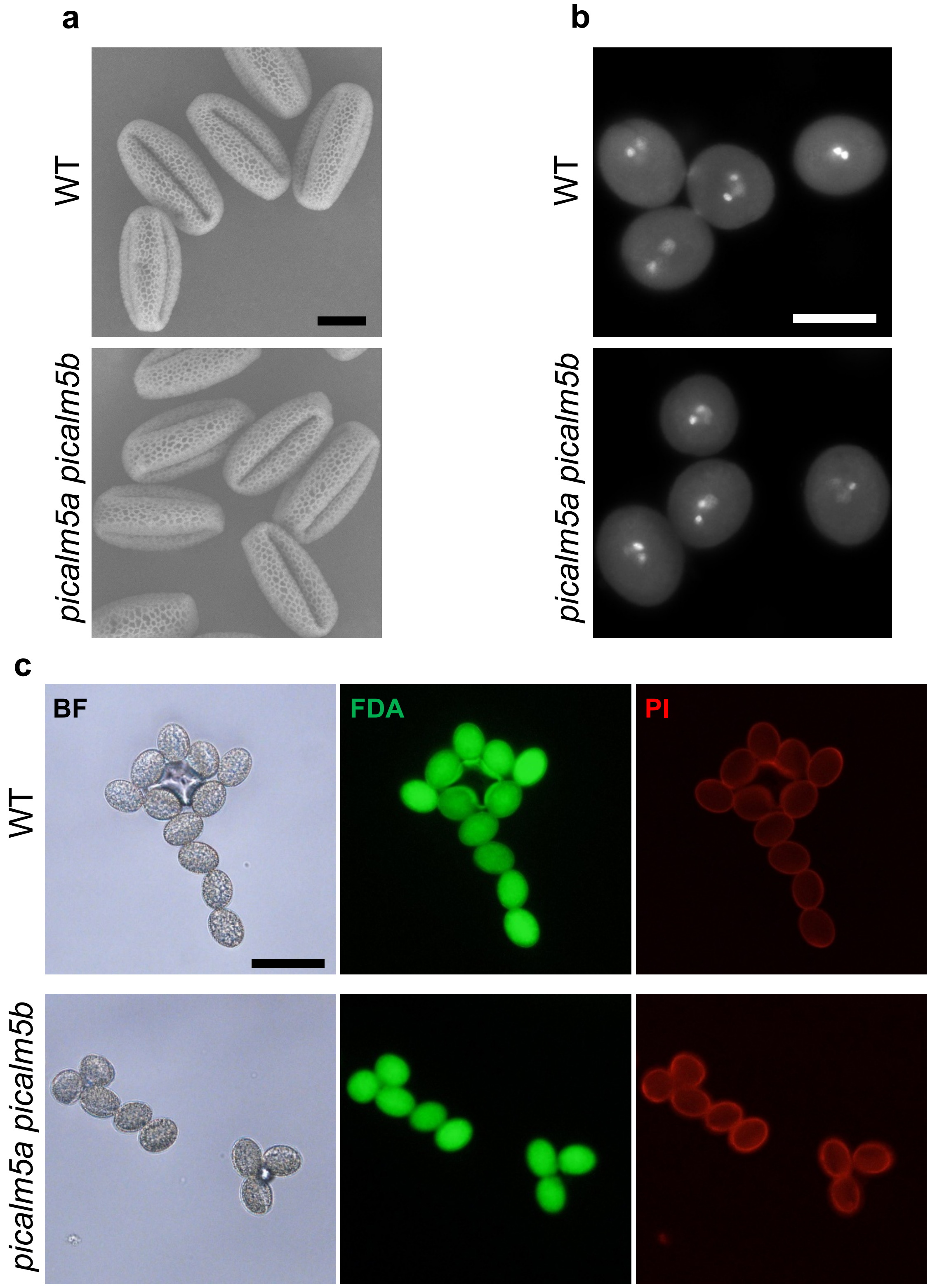

Supplementary Figure 2. Morphology and viability of *picalm5a picalm5b* mutant pollen grains. **a** Scanning electron micrographs of wild-type and *picalm5a picalm5b* pollen grains. Scale bar = 10 µm. **b** DAPI staining of wild-type and *picalm5a picalm5b* pollen. Scale bar = 20 µm. **c** PI/FDA staining of wild-type and *picalm5a picalm5b* pollen. FDA stains live pollen and PI stains dead pollen. Scale bar = 50 µm.

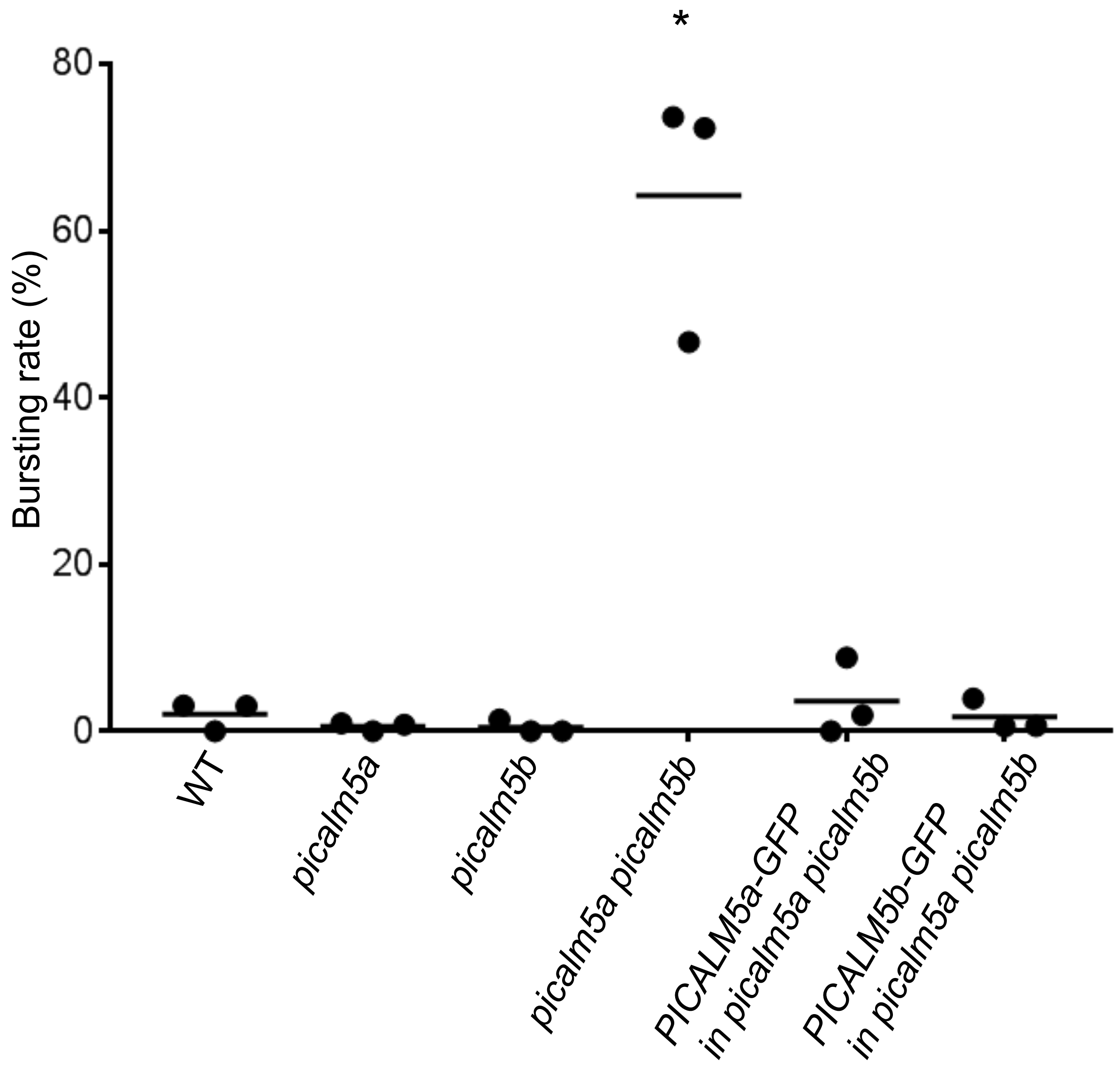

Supplementary Figure 3. Bursting rate of pollen tubes from *picalm5* mutants. Pollen tube tips were observed after 5-hour incubation on the solid pollen germination medium. Experiments were repeated three times (n = 97 ~ 170 for each experiment), and the means of each experiment are plotted. Bars and asterisk indicate means of three experiments and a significant difference from the wild type according to Welch's *t* test (p = 0.0196), respectively.

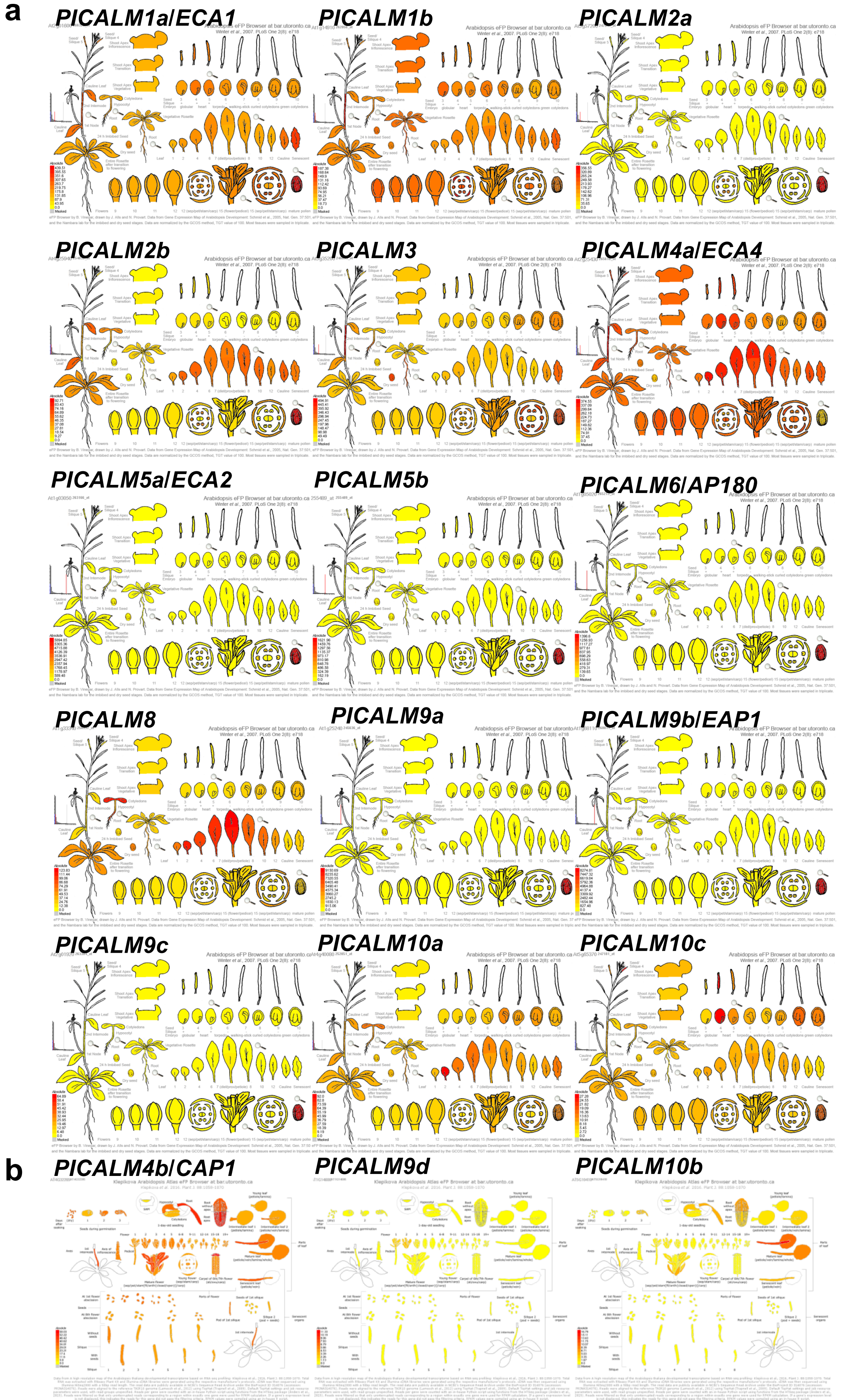

Supplementary Figure 4. Expression patterns of *PICALM* members in the public database. **a and b** Expression patterns based on microarray (**a**) and RNA-seq (**b**) data were collected from the Arabidopsis Electronic Fluorescent Pictograph (eFP) Browser[1-3] (http://bar.utoronto.ca/efp/cgi-bin/efpWeb.cgi).

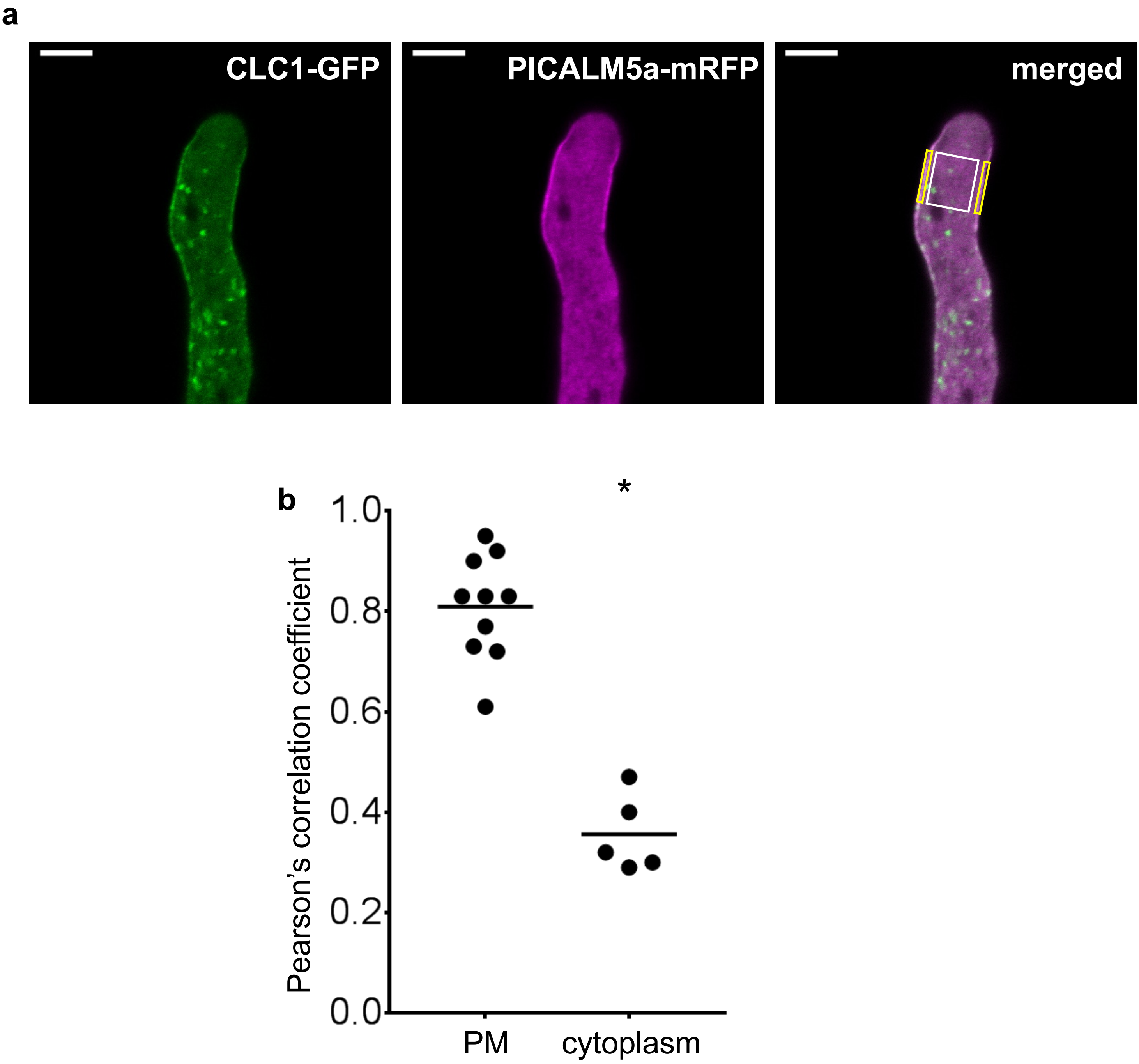

Supplementary Figure 5. Quantification of colocalization between CLC1-GFP and PICALM5a-mRFP. **a** An example of images used in the quantification analysis. The Pearson's correlation coefficient was calculated for the subapical cytoplasmic region (5 µm x 4 µm, white box) and subapical plasma membrane regions (5 µm x 0.5 µm, yellow box) in pollen tubes expressing CLC1-GFP and PICALM5a-mRFP using ImageJ with the PSC colocalization plugin. Scale bar = 5 µm. **b** The result of the colocalization analysis. Data were obtained from images of five pollen tubes (n = 10 for the plasma membrane and 5 for the cytoplasm) expressing CLC1-GFP and PICALM5a-mRFP. Bars represent means and the asterisk indicates significant difference from WT according to Student's t test ($p = 1.03 \times 10^{-6}$).

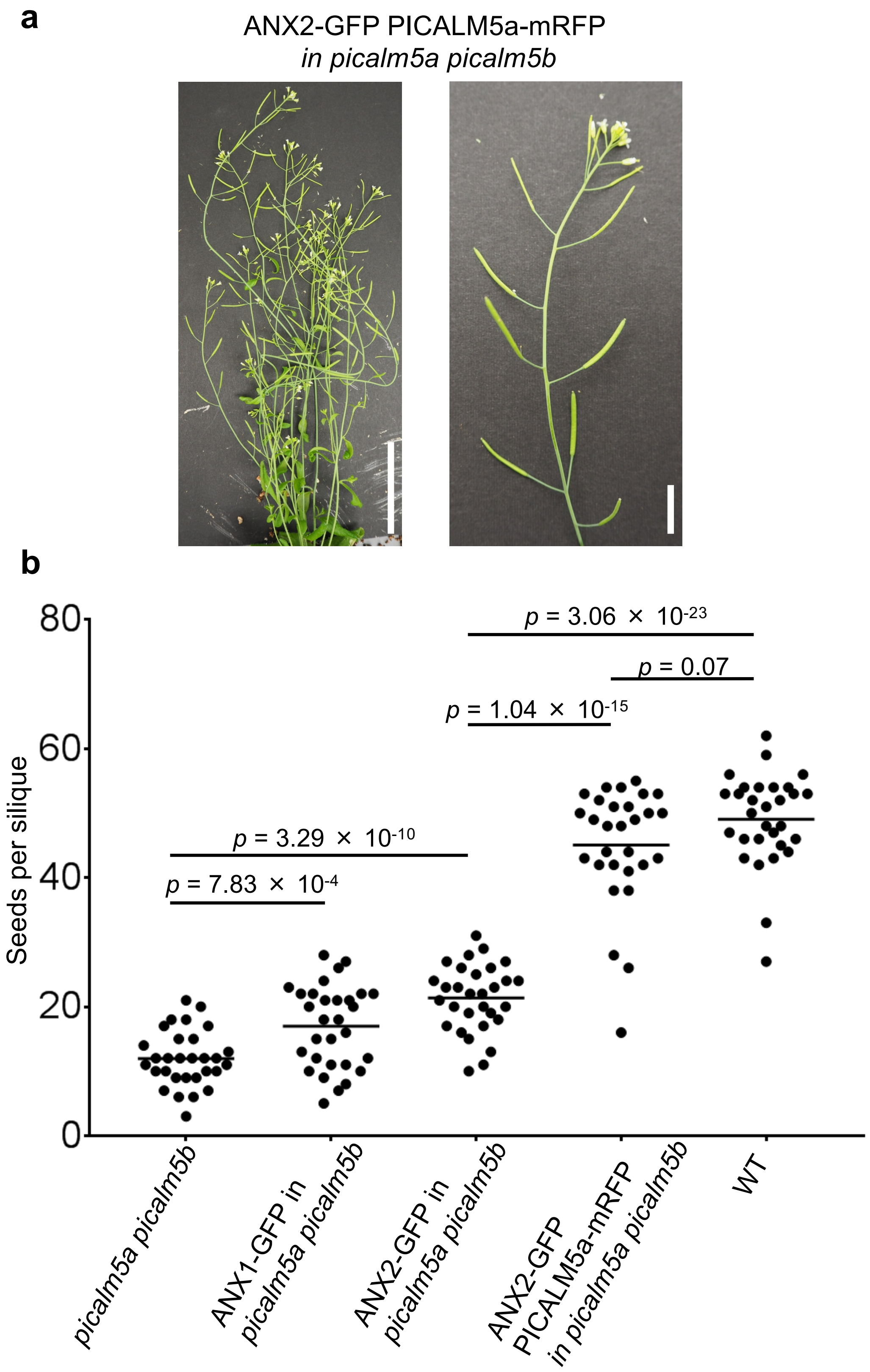

Supplementary Figure 6. Effect of ANXUR or PICALM5a expression on fertility. **a** The shoot and inflorescence of six-week-old in a *picalm5a picalm5b* plant expressing ANX2-GFP and PICALM5a-mRFP. Scale bars = 5 cm (shoot) and 1 cm (inflorescence). **b** Quantification of seeds per silique for *picalm5a picalm5b*, ANX1-GFP in *picalm5a picalm5b*, ANX2-GFP in *picalm5a picalm5b,* ANX2-GFP PICALM5a-mRFP in *picalm5a picalm5b*, and wild-type plants (n = 29 for ANX2-GFP PICALM5a-mRFP in *picalm5a picalm5b* and 30 for the others). The same data as Fig. 1d is displayed for *picalm5a picalm5b* and wild type. Bars represent means and p values of Welch's t test are presented.

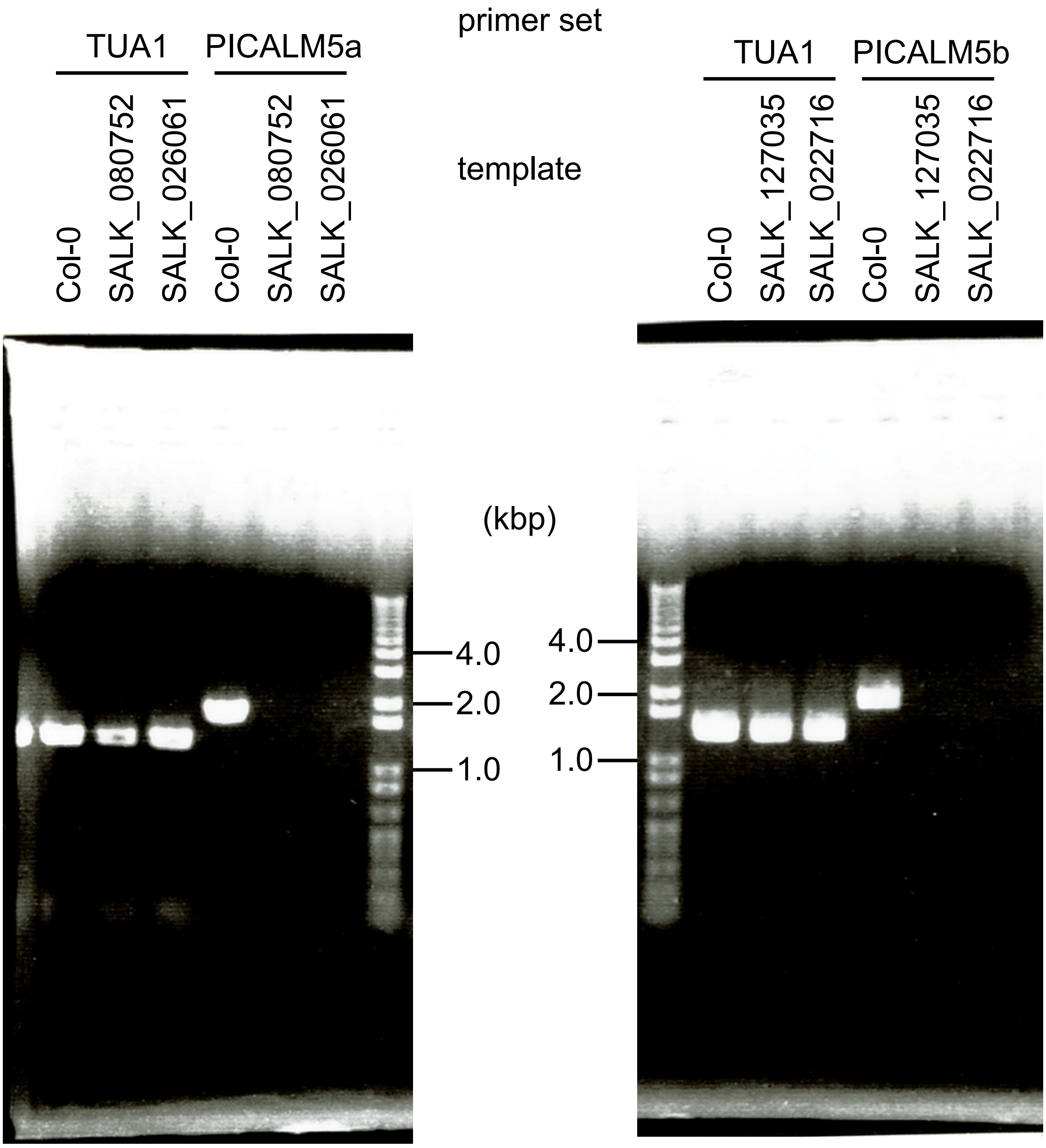

Supplementary Figure 7. Full scan of the gel images presented in Supplementary Fig. 1.

Supplementary Table 1. List of ANTH domain-containing proteins in Arabidopsis

Gene ID	Name	Other Name
At2g01600	PICALM1a	ECA1
At1g14910	PICALM1b	
At5g57200	PICALM2a	
At4g25940	PICALM2b	
At5g35200	PICALM3	
At2g25430	PICALM4a	ECA4
At4g32285	PICALM4b	CAP1
At1g03050	PICALM5a	ECA2
At4g02650	PICALM5b	
At1g05020	PICALM6	AP180
At1g33340	PICALM8	
At1g25240	PICALM9a	
At1g68110	PICALM9b	EAP1
At2g01920	PICALM9c	
At1g14686	PICALM9d	
At4g40080	PICALM10a	
At5g10410	PICALM10b	
At5g65370	PICALM10c	

Supplementary Table 2. List of primers used in this study

No.	Primer Name	Sequence (5'→3')
1	PICALM5a_CDS_for	CACCATGGGTTCGAGTAAGTTTAAACGAGCCATAG
2	PICALM5a_CDS_rev	ATATTGAGGTGTGTAAGAGTAAGGTTGGTTTTG
3	PICALM5b_CDS_for	CACCATGGGTTCAAGTAAGCTAAAACGTGCCATAG
4	PICALM5b_CDS_rev	ATAGCGAGGCGTGTAAGAGTAAGGTC
5	PICALM5a_genomic_for	CACCGACTGTTGATGCGGCATTGGTG
6	PICALM5a_genomic_rev	CTTGAACAGTCAAATTGTGTTGGCATG
7	PICALM5a_InFusion_for	TGATTGTGCCCCATAGAAGAAGCTC
8	PICALM5a_InFusion_rev	GCCACTACCTCCATATTGAGGTGTGTAAGAGTAAGGTTGGTTTTG
9	GFP_InFusion(PICALM5a)_for	TATGGAGGTAGTGGCATGGTGAGCAAGGGCGAGGAG
10	GFP_InFusion(PICALM5a)_rev	TATGGGGCACAATCATTACTTGTACAGCTCGTCCATGCCGTG
11	mRFP_InFusion(PICALM5a)_for	TATGGAGGTAGTGGCATGGCCTCCTCCGAGGAC
12	mRFP_InFusion(PICALM5a)_rev	TATGGGGCACAATCAGGCGCCGGTGGAGTG
13	PICALM5b_genomic_for	CACCCTTGAGACTGAGTGCGCTAC
14	PICALM5b_genomic_rev	GTCTTATGTTAATAACCTGTAGCCCACTC
15	PICALM5b_InFusion_for	TGATATTATATAAATAAACAATCACCATTAATGAAGAGCTTG
16	PICALM5b_InFusion_rev	GCCACTACCTCCATAGCGAGGCGTGTAAGAGTAAG
17	GFP_InFusion(PICALM5b)_for	TATGGAGGTAGTGGCATGGTGAGCAAGGGCGAGGAG
18	GFP_InFusion(PICALM5b)_rev	ATTTATATAATATCATTACTTGTACAGCTCGTCCATGCCGTG
19	SYP124_genomic_for	CACCAGTACCACGACAATTGCAAAATC
20	SYP124_genomic_rev	CACACATGTAGTTTTGATTACTAACTTG
21	SYP124_InFusion_for	AAGGGAGGTAGTGGCATGAATGATTTATTCTCTAGTTCGTTC
22	SYP124_InFusion_rev	TCGCCCTTGCTCACCATTTTTTTTCCTTTTTGCTTTTAAAAAGATTC
23	SYP125_genomic_for	CACCAGACTGCAACTTATGTGTTACCTAC
24	SYP125_genomic_rev	GGATTTAGCAGAAGAAAGTTTTG
25	SYP125_InFusion_for	AAGGGAGGTAGTGGCATGAACGATTTATTCTCTAATTCATTCAAG
26	SYP125_InFusion_rev	TCGCCCTTGCTCACCATTTTTACTCTGTCTTTATTTTCTTTCTCTG
27	SYP131_genomic_for	CACCCCAAAGAATCAGACTTTGTCTCAC
28	SYP131_genomic_rev	GCATCCACTTCCTGAATGC
29	SYP131_InFusion_for	AAGGGAGGTAGTGGCATGAACGACCTCTTAAAGGTTTG
30	SYP131_InFusion_rev	TCGCCCTTGCTCACCATTGTCGGAGAGAACGGGAACAG
31	GFP_InFusion(SYP)_for	ATGGTGAGCAAGGGCGAGGAG
32	GFP_InFusion(SYP)_rev	GCCACTACCTCCCTTGTACAGCTCGTCCATGCC
33	ANX1_genomic for	CACCCTTGTGCCCAATGTCTCAAC
34	ANX1_CDS-sc_rev	TCGTCCTTTGGGATTTACAATC
35	ANX2_genomic for	CACCGGCTTTGCTTCAGGGTCTAA
36	ANX2_CDS-sc_rev	TCGTCCTTTAGGGTTTACAATCT
37	CLC1_for	TTTAGGATCCACTAGTATGGCGACTTTTGATGATG
38	CLC1_rev	TGCTCACCATACTAGTGCCACTACCTCCCTC

Supplementary References

1. Schmid, M. *et al.* A gene expression map of Arabidopsis thaliana development. *Nat. Genet.* **37**, 501-506 (2005).

2. Winter, D. *et al.* An "Electronic Fluorescent Pictograph" browser for exploring and analyzing large-scale biological data sets. *PLoS One* **2**, e718 (2007).

3. Klepikova, A. V., Kasianov, A. S., Gerasimov, E. S., Logacheva, M. D. & Penin, A. A. A high resolution map of the Arabidopsis thaliana developmental transcriptome based on RNA-seq profiling. *Plant J.* **88**, 1058-1070 (2016).
